# Dexamethasone and potassium canrenoate alleviate hyperalgesia by competitively regulating IL‐6/JAK2/STAT3 signaling pathway during inflammatory pain in vivo and in vitro

**DOI:** 10.1002/iid3.721

**Published:** 2022-10-26

**Authors:** Jie Liu, Xiaolan Xie, Kai Qin, Le Xu, Juxiang Peng, Xiangyu Li, Xiongjuan Li, Zhiheng Liu

**Affiliations:** ^1^ Department of Anesthesiology The Second Affiliated Hospital of Guangzhou University of Chinese Medicine Guangzhou Guangdong China; ^2^ Department of Anesthesiology, Shenzhen Second People's Hospital The First Affiliated Hospital of Shenzhen University, Health Science Center Shenzhen Guangdong China

**Keywords:** dexamethasone, IL‐6, inflammatory pain, potassium canrenoate

## Abstract

**Background:**

Dexamethasone (Dexa) and potassium canrenoate (Cane) modulate nociceptive behavior via glucocorticoid receptor (GR) and mineralocorticoid receptor (MR) by two mechanisms (genomic and nongenomic pathways). This study was designed to investigate the Dexa‐ or Cane‐mediated nongenomic and genomic effects on mechanical nociception and inflammation‐induced changes in interleukin‐6 (IL‐6) mediated signaling pathway in rats.

**Methods:**

Freund's complete adjuvant (FCA) was used to trigger an inflammation of the right hind paw in male Sprague–Dawley rats. First, the mechanical nociceptive behavioral changes were examined following intraplantar administration of GR agonist Dexa and/or MR antagonist Cane in vivo. Subsequently, the protein levels of IL‐6, IL‐6Rα, JAK2, pJAK2, STAT3, pSTAT3^Ser727^, migration inhibitory factor, and cyclooxygenase‐2 were assessed by Western blot following intraplantar injection of Dexa or Cane or the combination. Moreover, the molecular docking studies determined the interaction between Dexa, Cane, and IL‐6. The competition binding assay was carried out using enzyme‐linked immunosorbent assays (ELISA).

**Results:**

Administration of Dexa and Cane dose‐dependently attenuated FCA‐induced inflammatory pain. The sub‐additive effect of Dexa/Cane combination was elucidated by isobologram analysis, accompanied by decrease in the spinal levels of IL‐6, pJAK2, and pSTAT3^Ser727^. The molecular docking study demonstrated that both Dexa and Cane displayed a firm interaction with THR^138^ binding site of IL‐6 via a strong hydrogen bond. ELISA revealed that Dexa has a higher affinity to IL‐6 than Cane.

**Conclusions:**

There was no additive or negative effect of Dexa and Cane, and they modulate the IL‐6/JAK2/STAT3 signaling pathway through competitive binding with IL‐6 and relieves hypersensitivity during inflammatory pain.

## Graphical abstract

FCA‐induced peripheral inflammatory pain cause central sensitization leading to the release of proinflammatory cytokine IL‐6, local intraplantar injection of Dexamethasone (Dexa) or Potassium canrenoate (Cane) competitively engage in IL‐6‐mediated IL‐6R/JAK2/STAT3 signaling pathway, in which Dexa was dominant in binding to IL‐6, thus regulating inflammatory pain. FCA, Freund's complete adjuvant; IL‐6, Interleukin 6; IL‐6R, Interleukin 6 receptor; JAK2, Janus kinase 2; STAT3, Signal transducer and activator of transcription 3;

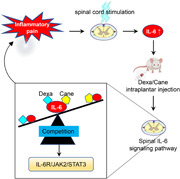

## 1 INTRODUCTION

Dexamethasone (Dexa) is an effective synthetic glucocorticoid acting primarily through the glucocorticoid receptor (GR) and is commonly used as a therapeutic agent that has anti‐inflammatory, antishock, and immunosuppressive effects.^1^ Potassium canrenoate (Cane) is a mineralocorticoid receptor (MR) antagonist, utilized as a diuretic in clinical practice for patients with liver disease, congestive heart failure, and chronic kidney disease.^2^ Both Dexa and Cane mediate the anti‐inflammatory via genomic effects by binding to nuclear receptor GR and MR, thus regulating the delay‐time of specific gene transcription.^3^ Recently, the classic genomic effects (usually take several hours or days to manifest) of GR and MR are significantly extended because of their rapid nongenomic mechanism (usually occur within a few minutes) via membrane‐bound receptors.^4^

Accumulating evidence suggested that GR and MR were involved in acute pain. Dexa (GR agonist) and Cane (MR antagonist) regulate the pain sensitization through rapid nongenomic effects by binding to neurons in dorsal root ganglia or spinal dorsal horn.^5^, ^6^ Moreover, both molecules rapidly attenuate pain behavior during rat hind paw inflammation in a dose‐dependent manner.^7^, ^8^ Interestingly, perineural Dexa is more efficacious than intravenous administration due to its direct action on nociceptive neurons.^9^ Previous studies reported that GRs are dominant on nociceptive neurons (peptidergic and nonpeptidergic nociceptor), whereas the expression of MRs dominates in peptidergic nociceptive neurons of normal rats.^5^, ^6^

Inflammation cascades upregulate multiple proinflammatory cytokines. Among these, interleukin‐6 (IL‐6) is a predominance of cytokines that binds to the IL‐6 receptor (IL‐6R) then activates the JAK/STAT signaling pathway.^10^ Another proinflammatory cytokine is macrophage migration inhibitory factor (MIF), which is released by activated cells after stimulation, altering the local inflammatory microenvironment by promoting the secretion of many kinds of proinflammatory cytokines (e.g., cyclooxygenase‐2 [Cox‐2]), thus regulating the process of inflammation.^11^, ^12^

However, whether local administration of Dexa and Cane exerts a synergistic effect via IL‐6/IL‐6R/JAK2/STAT3 axis and their correlation with MIF and Cox‐2 in pain regulation is yet to be elucidated. Previous studies have showed that MIF and Cox‐2 may act an upstream protein of IL‐6 signaling pathway to induce an inflammatory response.^13^, ^14^ Accordingly, we hypothesized that MIF and Cox‐2 might exert an effect on spinal IL‐6‐related pathway during inflammatory pain. Herein, we investigated whether rat hind paw inflammation induced by Freund's complete adjuvant (FCA) alters the mechanical hypersensitivity behavior and spinal IL‐6/JAK2/STAT3 pathway, as well as MIF and Cox‐2 expression via GR agonists (Dexa) and MR antagonists (Cane) or their combination during the inflammatory response. These findings could provide a new view for the nongenomic and genomic effects of pain regulation.

## 2 METHODS

### 2.1 Animals

The Second Affiliated Hospital of Guangzhou University of Chinese Medicine approved all animal experiments (No. 2019051). A total of 102 adult male healthy Sprague–Dawley rats (8–10 weeks old; 220–250 g body weight) were employed in our study. Rats were allowed to acclimatize to the environment for 1 week under the following conditions: temperature 22 ± 2°C, relative humidity 55 ± 5% with 12 h light–12 h dark cycle, and access to food and water ab libitum.

### 2.2 Induction of inflammatory pain

FCA (Sigma), containing heat‐killed *Mycobacterium tuberculosis* and paraffin oil at a concentration of 1 mg/ml, was used to induce inflammatory pain. Rats were injected 150 μl of FCA in the footpad of the right hind paw under inhalational anesthesia with 1–3% isoflurane (Baxter) and 1 L/min of oxygen flow rate,^7^ while the left hind paw remained intact. The physical symptoms (e.g., redness and swelling) and pain phenomenon (e.g., foot lifting, licking, and shaking) were observed after 24 h of FCA injection.

### 2.3 Reagents and experimental protocols

**Figure 1:**
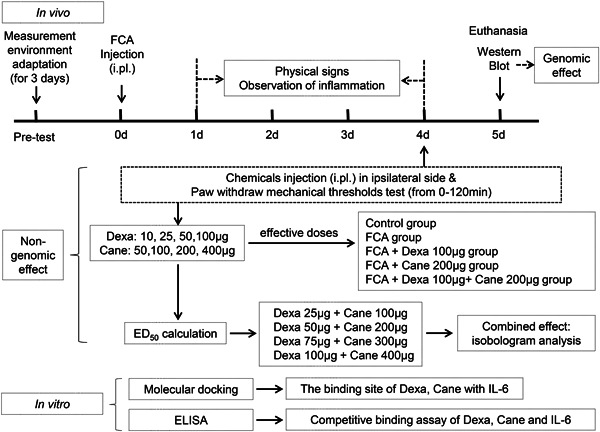
Schematic of the experimental protocol. Nongenomic effect (rapid): usually occur within a few minutes via membrane‐bound receptors. Genomic effect (delayed and long‐lasting): usually take several hours or days to manifest specific gene transcription. Cane, potassium canrenoate; Dexa, dexamethasone; ED_50_, median effective dose; ELISA, enzyme‐linked immunosorbent assay; FCA, Freund's complete adjuvant; IL‐6, interleukin 6; i.pl., intraplantar.

Dexa and Cane were obtained from ApexBio and TargetMol, respectively. The experimental protocol is illustrated in Figure 1. First, we examined the impact of intraplantar injection of increasing doses of GR agonist Dexa (10, 25, 50, 100 μg/100 μl) and MR antagonist Cane (50, 100, 200, 400 μg/100 μl) in a blinded fashion on paw withdraw mechanical threshold (PWMT). Dexa was solubilized in 1% dimethylsulfoxide with phosphate buffered saline (PBS), and Cane was dissolved with PBS. We determined the anti‐hyperalgesia median effective dose (ED_50_) according to the dose‐dependent curve. Then, the concomitant intraplantar injection of Dexa/Cane combination (Dexa 25 μg + Can 100 μg/100 μl, Dexa 50 μg + Can 200 μg/100 μl, Dexa 75 μg + Can 300 μg/100 μl, Dexa 100 μg + Can 400 μg/100 μl) was administrated at the calculated of ED_50_ and determined the combination of Dexa/Cane by isobologram analysis. PWMTs were either in grams or maximum possible effect percentage (MPE%) (MPE = PWMT_postinjection_ − PWMT_baseline_/18 − PWMT_baseline_). Then we determined the values before and 15, 30, 60, and 120 min after drug administration. The effective doses of each drug were chosen for subsequent steps.

Second, rats were randomly divided into 5 groups (*n* = 6/group) based on the previous experiments as follows: (1) Naïve rats (Control group: no treatment); (2) FCA group; (3) FCA + Dexa 100 μg/100 μl (Dexa group); (4) FCA + Cane 200 μg/100 μl (Cane group); (5) FCA + Dexa 100 μg + Cane 200 μg/100 μl (Dexa + Cane group). The treatment group received a drug injection on day 4. Subsequently, the rats were killed on day 5 following FCA injection to test the spinal level of MIF, Cox‐2, and IL‐6 signaling pathway‐related proteins. Finally, the molecular docking and enzyme‐linked immunosorbent assay (ELISA) were used to analyze the binding of Dexa, Cane, and IL‐6 in vitro.

### 2.4 Von Frey test

Von Frey filaments (Stoelting) were used to access the alteration in mechanical sensitivity after FCA‐induced inflammatory pain. Rats were placed in a wire mesh floor for at least 1 h for 3 days before the experiments. The baseline levels of animals were tested before drug application using Von Frey filament test with up‐and‐down method.^15^ The 50% response threshold was determined using the following formula: 50% g threshold = (10^(xf + kδ)^)/10,000, according to the resulting pattern of positive and negative modes (X = withdrawal and 0 = no withdrawal). Finally, the area under the receiver operating characteristic curve (AUC) was applied to calculate and determine the effects of chemical injection.

### 2.5 Western blot

Rats were euthanized by anesthetic over‐dose with isoflurane inhalation. The lumbar spinal cord (L3–5) were collected and homogenized to obtain protein extract for Western blot analysis. Protein extract was centrifuged at 16,000*g* for 15 min at 4°C.

The equal quantified proteins (50 μg) were denatured and loaded for separation by 10%–12% sodium dodecyl sulfate–polyacrylamide gel electrophoresis, then transferred to the polyvinylidene fluoride membrane (Millipore). Membranes were blocked with 5% nonfat milk/TBST for 2 h at room temperature and probed with the following primary antibodies: rabbit anti‐IL‐6, anti‐IL‐6Rα, anti‐JAK2, anti‐pJAK2, anti‐STAT3, anti‐pSTAT3, anti‐MIF, anti‐Cox‐2, and anti‐β‐actin (1:1000; Affinity). Subsequently, the membranes were incubated with 1:10,000 horseradish peroxidase‐conjugated goat anti‐rabbit secondary antibody (Affinity) in 5% nonfat milk/TBST for 1 h at room temperature. Finally, proteins were visualized with ECL reagent (Affinity) and analysed by ImageJ software (National Institutes of Health, Bethesda, MD, USA).

### 2.6 Molecular docking

The molecular docking of Dexa and Cane with IL‐6 were performed using AutoDock 4.2 package, as described previously.^16^ The steps were as follows: (1) Ligand preparation, determine the molecular weight and 2D structure of Dexa (CID: 5743) and Cane (CID: 656615) from the PubChem database. Open Babel GUI software was used to convert 2D format to 3D mol2 format. (2) Molecule preparation, the 3D structure of IL‐6 (PDB ID: 1ALU, in *PDB format) was downloaded from the RCSB Protein Data Bank. Discovery Studio software was employed to remove all crystal waters and the original ligand conformation of IL‐6 and then save it in *PDB format. (3) Molecular docking, the docking simulations were performed for the structure of Dexa and Cane with AutoDock Tools (version 1.5.6). (4) Data outputs, the visualization of the docking analysis was rendered by PyMol. The 2D diagram of protein‐ligand interactions was performed by Discovery Studio software.

### 2.7 Competition binding assay by ELISA in vitro

The competition binding assay between Dexa, Cane, and IL‐6 was performed using ELISA method. Competing IL‐6 (0–2000 pg/ml) was mixed with 1 mM Dexa (1 mg/2.548 ml) and/or 1 mM Cane (1 mg/2.522 ml) and incubated at 37°C for 1 h. Then, the concentration of IL‐6 in the presence of 1 mM Dexa and/or Cane was determined using rat IL‐6 ELISA kit (Elabscience), according to the operating instruction.

### 2.8 Statistical analysis

All data were presented as mean ± standard deviation (*SD*). The alterations between different drug doses and time intervals using two‐way analysis of variance (ANOVA), followed by Bonferroni test. Linear regression analysis assessed the dose‐dependent and the combined effects. The data from the Western blot and competition binding assay were analyzed by one‐way ANOVA, followed by the Bonferroni test for comparison. SPSS 19.0 (IBM) was used for statistical analyses, and graphs were plotted using GraphPad Prism 7.0 software (GraphPad Software Inc.). All *p* values <.05 indicated statistical significance.

## 3 RESULTS

### 3.1 Dexa versus cane mediated nociception in rats with ongoing inflammatory pain

**Figure 2:**
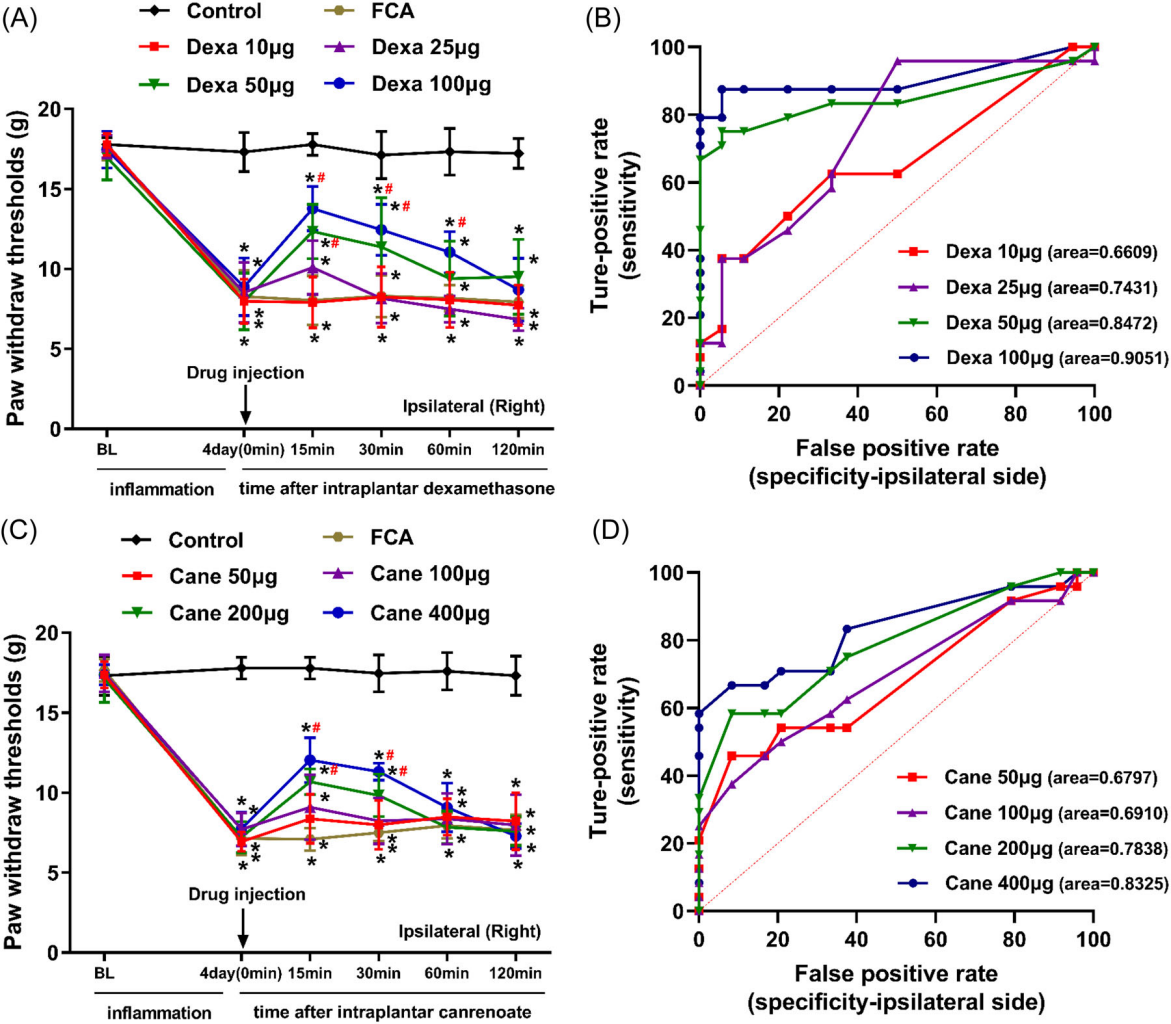
Role of GR agonist dexamethasone (Dexa) and MR antagonist potassium canrenoate (Cane) in the modulation of inflammation‐induced nociceptive behavior in the right hind paw. (A and C) Four days of Freund's complete adjuvant (FCA) of the right hind paw resulted in dose‐dependently reduced paw withdraw mechanical threshold (PWMT) compared to baseline values (0 min). Increasing doses of intraplantar Dexa (A) or Cane (C) reversed the already reduced mechanical hypersensitivity within minutes (*p* < .05; two‐way ANOVA, Bonferroni test, *n* = 6). Both Dexa and Cane show a transient attenuation via GR and MR during rat hind paw inflammation. Data are expressed as mean ± *SD*. BL, baseline. (B and D) ROC curves and AUC values of Dexa and Cane in the ipsilateral side of FCA‐induced inflammatory pain. ANOVA, analysis of variance; AUC, area under the receiver operating characteristic curve; GR, glucocorticoid receptor; MR, mineralocorticoid receptor; ROC, receiver operating characteristic curve.

A significant decrease was noted in the mechanical pain threshold following the administration of FCA. The FCA‐induced mechanical hypersensitivity was reversed by intraplantar injection of GR agonist Dexa or the MR antagonist Cane in a dose‐dependent manner in the inflammatory hind paw (*p* < .05, Figure 2A and 2C). The intraplantar injection of both Dexa and Cane did not have any significant effects on the noninflamed side (*p* > .05, data not shown).

In rats with FCA‐induced inflammatory pain, the mean (*SD*) AUCs for increasing doses of Dexa (10, 25, 50, 100 μg) were 0.6609 (0.0840), 0.7431 (0.0793), 0.8472 (0.0636), and 0.9051 (0.0514), respectively (Figure 2B), and the mean (*SD*) AUCs for increasing doses of Cane (50, 100, 200, 400 μg) were 0.6797 (0.0782), 0.6910 (0.0767), 0.7838 (0.0661), and 0.8325 (0.0605), respectively (Figure 2D).

**Figure 3:**
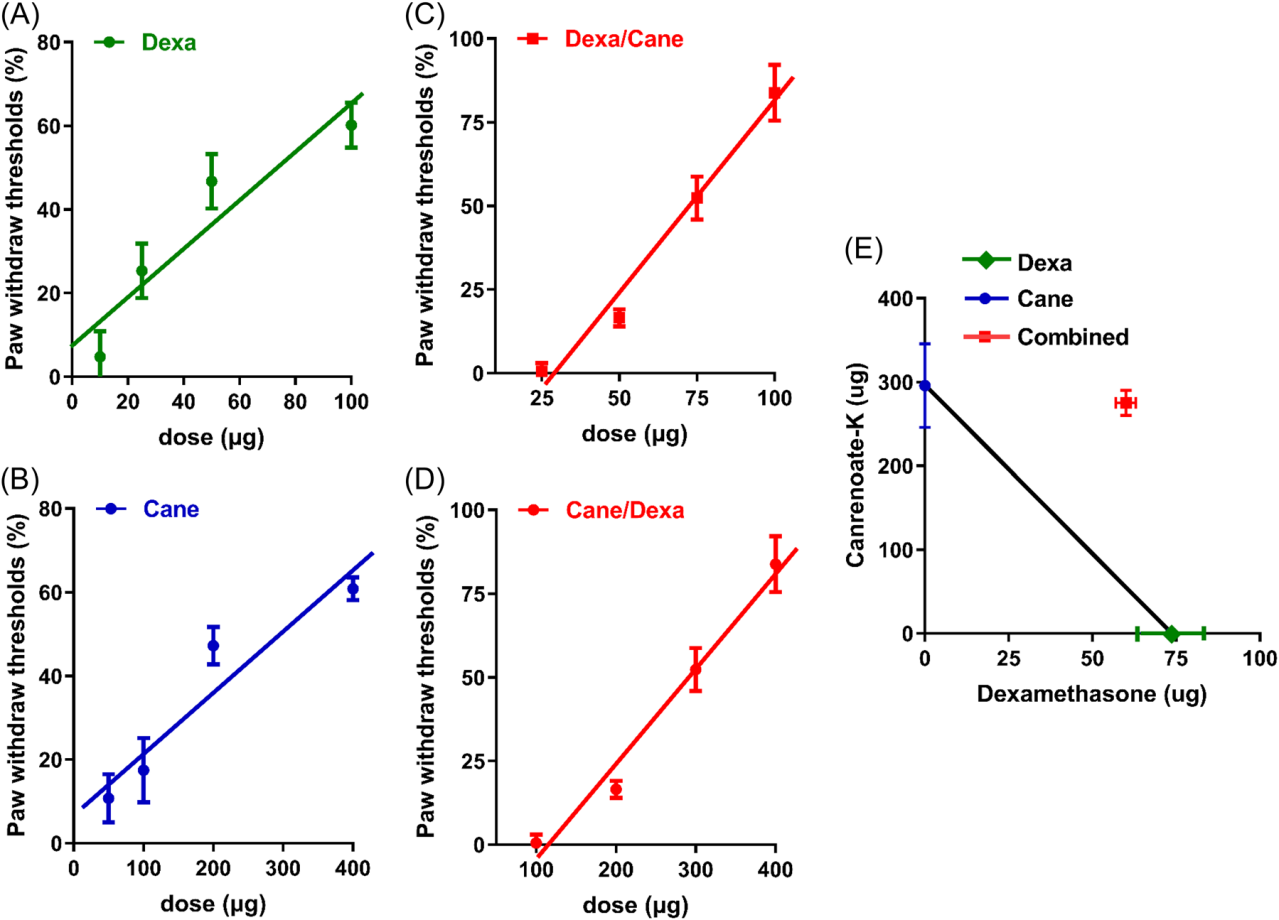
Nociceptive effects of the GR agonist Dexamethasone (Dexa) and MR antagonist potassium canrenoate (Cane) and their combination. (A and B) Dose‐effect curves of Dexa or Cane alone showed that the median effective dose (ED_50_) ratio was 73.38 μg and 295.62 μg, respectively. (C and D) Dose‐effect curves of Dexa and Cane in combination showed a rightward shift. (E) Isobologram analysis demonstrated that the point representing the ED_50_ of the combination of Dexa/Cane (red) was outside the line connecting the ED_50_ of Dexa alone (green) and Cane (blue), indicating a sub‐additive interaction of both drugs. Data are expressed as mean ± *SD* (*n* = 6). Statistical analysis was conducted using Linear regression. GR, glucocorticoid receptor; MR, mineralocorticoid receptor.

Furthermore, intraplantar administration of Dexa or Cane resulted in a dose‐dependent increase in PWMT in the ipsilateral side (Figure 3A,B). These dose‐response curves were shifted to the right when combined with the ED_50_ of the other drug (according to the Dexa/Cane ED_50_ ratio of 73.38 μg: 295.62 μg = 1:4.03, respectively, or vice versa) (Figure 3C,D). The combination resulted in a sub‐additive effect (neither a synergistic nor an additive effect) in the isobologram analysis (Figure 3E).

### 3.2 Changes in the IL‐6 signaling‐related protein following Dexa and Cane injection in vivo

**Figure 4:**
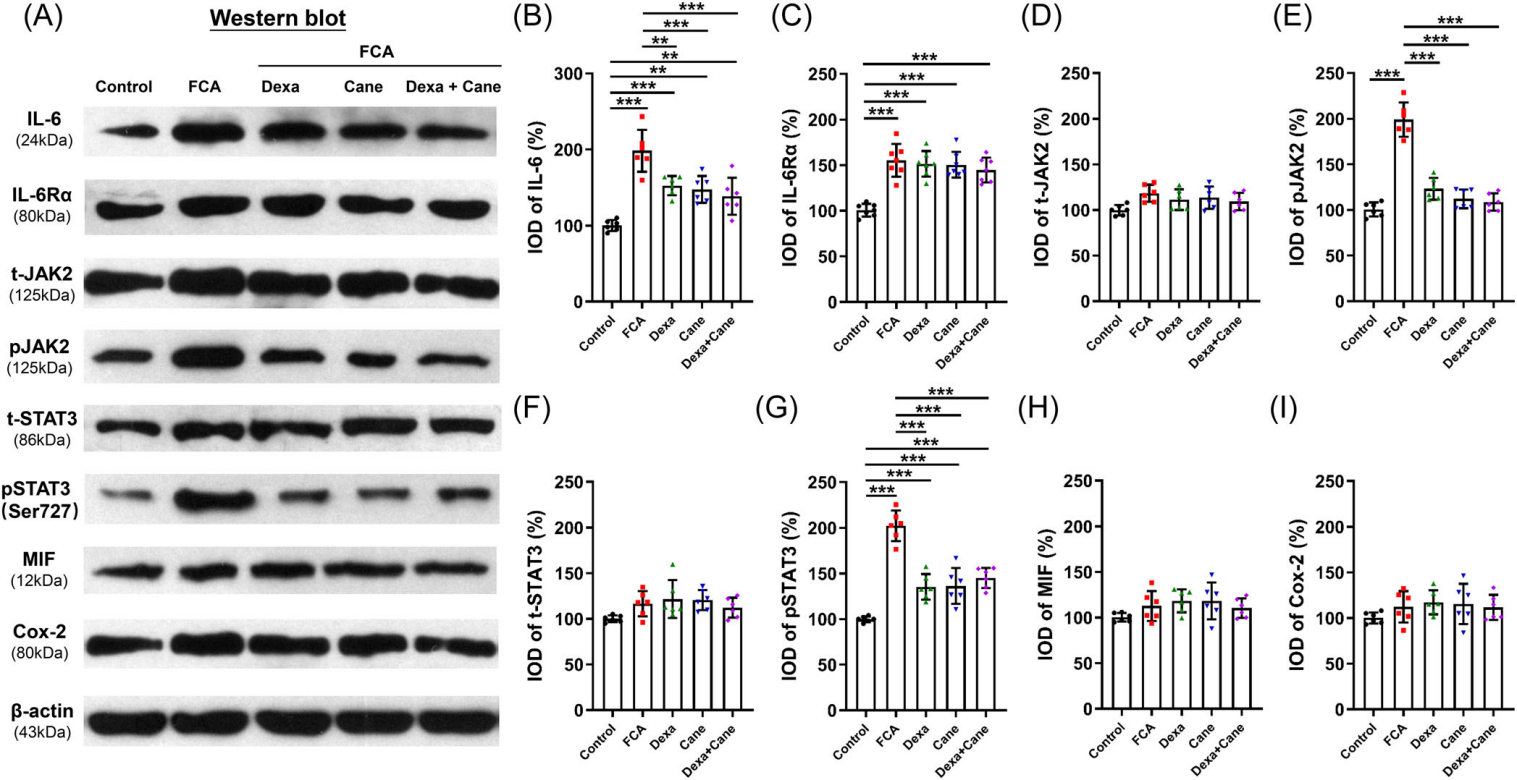
IL‐6‐induced signaling pathway and inflammation‐related protein changes were assessed in the spinal cord by Western blotting. (A–I): Levels of IL‐6, IL‐6Rα, JAK‐2, pJAK‐2, STAT3, pSTAT3 (Ser727), MIF, and Cox‐2 proteins in the spinal cord following intraplantar injection of Dexa (100 μg), Cane (200 μg), and the combination (Dexa 100 μg + Cane 200 μg). After intraplantar injection of Dexa and/or Cane, the IL‐6, pJAK2, pSTAT3 protein expression levels were reduced in FCA‐treated rats, and STAT3, MIF, Cox‐2 did not change between groups (*p* > .05). Values were normalized against GAPDH and expressed as a percentage of control. Data are expressed as mean ± *SD* (*n* = 6). *p < .05; **p < .01; ***p < .001. Statistical comparisons were conducted using one‐way ANOVA with Bonferroni's test. ANOVA, analysis of variance; Cane, potassium canrenoate; Cox‐2, cyclooxygenase‐2; Dexa, dexamethasone; FCA, Freund's complete adjuvant; IL‐6, interleukin‐6; IL‐6Rα, interleukin 6 receptor α; IOD, integrated optical density; JAK2, Janus kinase 2; MIF, macrophage migration inhibitory factor; STAT3, Signal transducer and activator of transcription 3.

In the spinal cord sections, Dexa and Cane significantly decreased the IL‐6, pJAK2, and pSTAT3^Ser727^ protein levels, which did not decrease further by Dexa/Cane combination (Figure 4A,B, 4E, 4G), while the levels of IL‐6Rα, total JAK2, total STAT3, MIF, and Cox‐2 did not show any difference among groups following FCA‐induced inflammatory pain (Figure 4C,D, 4F, 4H,I).

### 3.3 Molecular docking of Dexa and Cane with IL‐6

**Figure 5:**
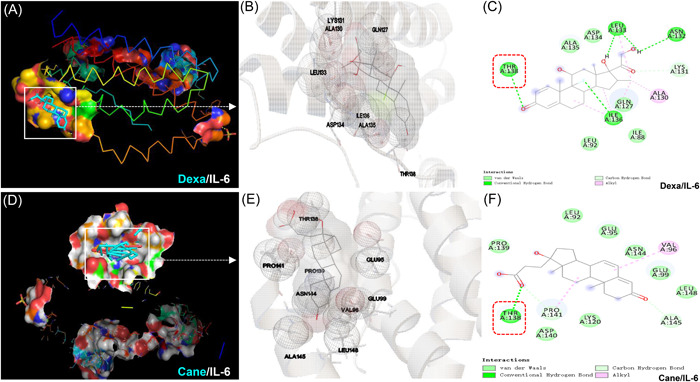
Molecular docking of IL‐6 to Dexamethasone (Dexa) or potassium canrenoate (Cane). (A and D) Docked model of IL‐6 (in carton mode) bound to Dexa or Cane (indicated by cyan in white boxes) with a binding energy of −7.21 kcal/mol and −6.98 kcal/mol, respectively. (B and E) Magnified view of the docked structure showing the contact between IL‐6 and Dexa (B) or Cane (E), respectively. (C and F) 2D ligand‐molecular interaction diagram. Both Dexa (C) and Cane (F) interact with the residues of binding sites of IL‐6, and both competitively bind to THR^138^ binding site by the formation of the hydrogen bond. IL‐6, interleukin‐6.

In the analysis of molecular docking, we confirmed the binding site of Dexa and Cane with IL‐6. Figure 5A–C illustrate that Dexa forms a hydrogen bond with THR^138^, LEU^133^, ASN^132^, ILE^136^, and LYS^131^ of IL‐6 and interacts through *van der Waals* forces with ALA^135^, ASP^134^, LEU^92^, and ILE^88^. Figure 5D–F show that Cane interacts through THR^138^, PRO^141^, and ALA^145^ by the formation of hydrogen bond and interacts via van der Waals forces with PRO^139^, LEU^92^, GLU^95^, ASN^144^, GLU^99^, LEU^148^, LYS^120^, and ASP^140^ of IL‐6.

### 3.4 Competitive binding assay between Dexa, Cane, and IL‐6

**Figure 6:**
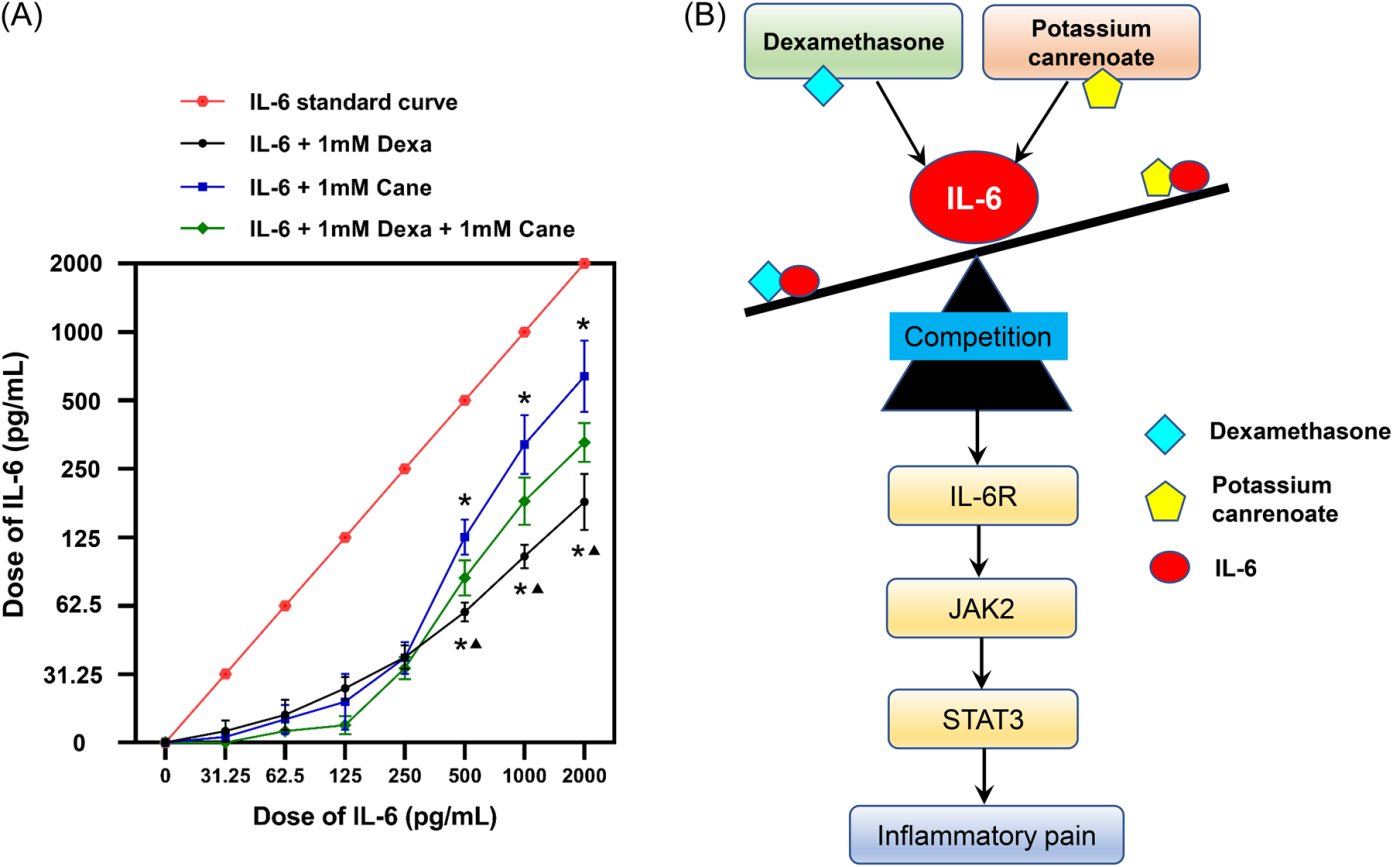
Competitive binding assay and the schematic summarizing the key findings of this study. (A) Indicated concentrations of competing IL‐6 (0–2000 pg/ml) were incubated with 1 mM Dexa and/or Cane at 37°C for 1 h. After incubation, the competitive binding of Dexa and/or Cane to IL‐6 was evaluated by competitive ELISA. The current data showed that Dexa had a higher affinity to IL‐6 than Cane at the same concentration. Data are expressed as mean ± *SD* (*n* = 6). *p < .05 versus IL‐6 + 1 mM Dexa + 1 mM Cane group; ^▲^ *p* < .05 versus IL‐6 + 1 mM Cane group. Statistical comparisons were conducted with one‐way ANOVA followed by Bonferroni's test. (B) Dexa and Cane competitively engage in IL‐6‐mediated JAK2/STAT3 signaling pathway, in which Dexa was dominant in binding to IL‐6, thus regulating tactile allodynia during inflammatory pain development. Cane, potassium canrenoate; Dexa, dexamethasone; ELISA, enzyme‐linked immunosorbent assays; IL‐6, interleukin‐6; IL‐6R, interleukin‐6 receptor; JAK2, Janus kinase 2; STAT3, Signal transducer and activator of transcription 3.

As shown in Figure 6A, the curve shifted downwards, indicating that the concentration of IL‐6 decreased in the presence of Dexa and/or Cane. In the concentration range of 500–2000 pg/ml, the level of IL‐6 cytokine was further decreased by Dexa than Cane administration, and the effect of Dexa and Cane combination was placed in the middle, demonstrating that Dexa was more potent than Cane in competition with the IL‐6 cytokine.

## 4 DISCUSSION

In the current study, we first examined the nongenomic effects (normally happen rapidly or within a few minutes) of Dexa or Cane in 120 min by using von Frey filaments, and their genomic effects (usually take place 6 h or days) on the spinal IL‐6 signaling pathway after 24 h were then investigated using Western blot. We found that Dexa (GR agonist) and Cane (MR antagonist) attenuated FCA‐induced nociceptive behavior via IL‐6 signaling pathway. As shown in the summarized mechanisms in Figure 6B, Dexa and Cane competitively bind to IL‐6, regulating the IL‐6/JAK2/STAT3 signaling pathway, thus influencing the sensitivity during inflammatory pain development.

Herein, we used an inflammatory pain model of unilateral FCA injection and local intraplantar injection of Dexa or Cane in rats. Local intraplantar administration of FCA leading to central sensitization following the activation of spinal astrocytes and microglia in rats.^7^ The PWMT was significantly reduced, and physical signs (redness and swelling) were observed after FCA injection. It has been documented that a local injection of steroid caused a high concentration of drug in the tissues and exert that is more effective than systemic application, indicating significant treatment effects and clinical benefit.^9^, ^17^ A previous study revealed that intraplantar administration of IL‐6 leads to mechanical hypersensitivity, as assessed by von Frey testing.^18^ It was additionally reported that the peripheral inflammation can cause central sensitization that can lead to the release of proinflammatory cytokines and chemokines in the spinal cord and brain, which play a powerful neuromodulators in inducing hyperalgesia and allodynia.^19^, ^20^ Therefore, it would be interesting to investigate whether intraplantar application of Dexa and/or Cane exhibits functional effects on the IL‐6‐induced signaling pathway in the spinal cord during the inflammatory process.

The current data showed that FCA‐induced inflammatory pain was alleviated by local administration of Dexa or Cane in a dose‐dependent manner within 60 min (Figure 2), suggesting rapid nongenomic effects. Inconsistent with a previous study,^21^ our data (Figure 3) indicated that the anti‐hyperalgesia ED_50_ of GR agonist Dexa combined with the ED_50_ of MR antagonist Cane showed a sub‐additive effect but not a synergistic or an additive effect according to the isobologram analysis.^22^ This phenomenon could be explained by the putative crosstalk between GR/MR and the downstream signaling pathways, which is consistent with previous reports, wherein GR colocalized with MR in neurons.^7^ Next, we detected the effect of Dexa and/or Cane on spinal IL‐6‐induced signaling pathway. We found that local intraplantar injection of Dexa or Cane decreases the levels IL‐6, pJAK2, and pSTAT3^Ser727^ proteins in the spinal cord (Figure 4), which did not decrease further by Dexa/Cane combination, suggesting crosstalk with the IL‐6/JAK2/STAT3 signaling pathway. This was in line with the crosstalk between GR and IL‐6R and shared the same downstream signaling pathway in the spinal cord.^23^ We selected intraplantar delivery of Dexa and/or Cane in effective doses to the rats and determined the protein in the spinal cord in the present study, the reasons could be as follows. (1) Direct effect: local administration of steroid hormones that are absorbed into the blood and pass through the blood brain barrier into the central nervous system,^24^ which may contribute to the reduction of cytokine levels by suppression of peripheral and central sensitization. (2) Indirect effect: peripheral inflammation with resulting nociceptive input (e.g., FCA‐induced hind paw inflammation) leads to the increased release of proinflammatory cytokines,^20^ and peripheral administration of drugs with analgesic potency can reduce proinflammatory cytokines and suppress nociceptive sensitization that occurs both at the peripheral and central level.^25^

Additionally, the molecular docking analysis displayed a possible interaction of Dexa or Cane with IL‐6 via hydrogen bond and van der Waals forces, and both Dexa and Cane could bind to the THR^138^ site of the IL‐6 by a hydrogen bond (Figure 5), demonstrating that these small molecules can form stable complexes and compete with IL‐6. Thus, it could be suggested that Dexa and Cane in combination with IL‐6 affect the IL‐6‐induced signaling pathway by decreasing the phosphorylation of JAK2 and STAT3, which in turn may down‐regulate the generation of IL‐6 via a positive feedback loop.^26^

Although several findings identified a correlation between GR, MR, and IL‐6 in inflammation and pain regulation,^27^, ^28^, ^29^ for the first time, we investigated the docking pattern of Dexa, Cane, and IL‐6, providing a putative binding site by molecular docking analysis. Notably, the molecular docking results are supported by the in vitro results, ELISA data showed that Dexa and Cane competitively bind to IL‐6 (Figure 6A), and Dexa shows a higher affinity to IL‐6 than Cane within a certain concentration range, indicating that both molecules might share the cellular machinery to regulate the IL‐6‐induced signaling pathway. Thus, the sub‐additive effects of Dexa/Cane combination in isobologram analysis and molecular docking results support the above theory.

The limitation of this study was that we focused only on IL‐6 cytokine, the clinical relevance requires further confirmation. Several other molecular mechanisms underlying GR agonist and MR antagonist analgesic effects are associated with various cytokines, including tumor necrosis factor‐α and IL‐1β.^27^, ^30^, ^31^, ^32^ However, an approach targeting IL‐6 has been applied in the treatment of various chronic inflammatory diseases.^33^ Therefore, further in‐depth elucidation of the regulation of IL‐6 signaling is of theoretical and clinical significance to pain management. Another limitation of this study lies in the fact that we did not check the actual concentration of Dexa or Cane in the spinal cord tissue after the drug injection. Thus, we cannot determine whether the analgesia effect of Dexa and/or Cane is direct or indirect in the spinal cord level. Nevertheless, our study provides further understanding of the role of GR agonist and MR antagonist in the IL‐6‐induced signaling pathway during inflammatory pain.

## 5 CONCLUSIONS

In summary, we demonstrated that there was no additive or negative effect of simultaneously pharmacological treatment of GR agonist dexamethasone and MR antagonist potassium canrenoate, and they competitively bind to IL‐6 cytokine, thus regulating the IL‐6/JAK2/STAT3 signaling pathway and relieving nociceptive behavior during FCA‐induced inflammatory pain.

## AUTHOR CONTRIBUTIONS

**Jie Liu**: performed the execution, data curation, and writing and editing of the original draft. **Xiaolan Xie, Kai Qin, Le Xu, Juxiang Peng**: collected the data and performed statistical analyses. **Xiangyu Li, and Zhiheng Liu**: reviewed the manuscript. **Xiongjuan Li**: designed the experiments, supervised, reviewed, and revised the manuscript. All authors read and approved the final manuscript.

## CONFLICTS OF INTEREST

The authors declare no conflicts of interest.

## ETHICS STATEMENT

This study was approved by the Institutional Animal Care and Use Committee at The Second Affiliated Hospital of Guangzhou University of Chinese Medicine.

## Data Availability

All data generated or analyzed during this study are included in this published article.
