# Correction to “Dexamethasone and Potassium Canrenoate Alleviate Hyperalgesia by Competitively Regulating Il‐6/JAK2/STAT3 Signaling Pathway During Inflammatory Pain In Vivo and In Vitro”

**DOI:** 10.1002/iid3.70197

**Published:** 2025-04-18

J. Liu, X. Xie, K. Qin, L. Xu, J. Peng, X. Li, X. Li and Z. Liu. “Dexamethasone and potassium Canrenoate Alleviate Hyperalgesia by Competitively Regulating Il‐6/JAK2/STAT3 Signaling Pathway During Inflammatory Pain In Vivo and In Vitro,” *Immunity, Inflammation and Disease* 10, no. 11 (2022): e721, https://doi.org/10.1002/iid3.721.

In Figure 4, the immunoblot band of Cox‐2 (Figure 4A) and the bar chart of Cox‐2 (Figure 4I) are incorrect. The correct Figure 4 is presented below.

**Figure 4:**
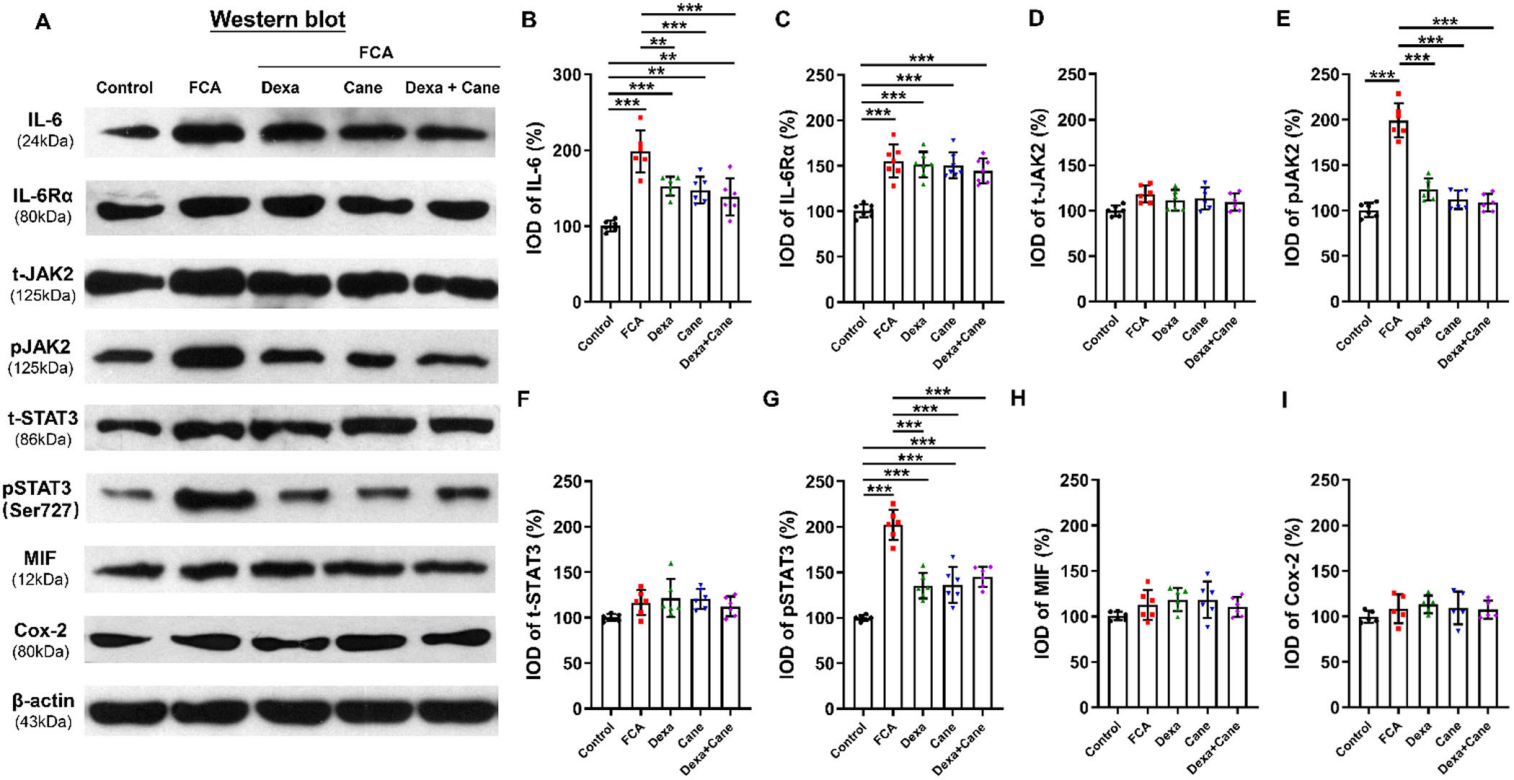
.

**Corrected** Figure 4

Additionally, the legend for Figure 4 is corrected (change is shown in bold):

IL‐6‐induced signaling pathway and inflammation‐related protein changes were assessed in the spinal cord by Western blotting. (A–I): Levels of IL‐6, IL‐6Rα, JAK‐2, pJAK‐2, STAT3, pSTAT3 (Ser727), MIF, and Cox‐2 proteins in the spinal cord following intraplantar injection of Dexa (100 μg), Cane (200 μg), and the combination (Dexa 100 μg + Cane 200 μg). After intraplantar injection of Dexa and/or Cane, the IL‐6, pJAK2, pSTAT3 protein expression levels were reduced in FCA‐treated rats, and STAT3, MIF, Cox‐2 did not change between groups (*p* > 0.05). Values were normalized against GAPDH and expressed as a percentage of control. Data are expressed as mean ± SD (***n* = 5–6**). *p < 0.05; **p < 0.01; ***p < 0.001. Statistical comparisons were conducted using one‐way ANOVA with Bonferroni's test. ANOVA, analysis of variance; Cane, potassium canrenoate; Cox‐2, cyclooxygenase‐2; Dexa, dexamethasone; FCA, Freund's complete adjuvant; IL‐6, interleukin‐6; IL‐6Rα, interleukin 6 receptor α; IOD, integrated optical density; JAK2, Janus kinase 2; MIF, macrophage migration inhibitory factor; STAT3, Signal transducer and activator of transcription 3.

The corrections do not change the results and conclusions. The authors apologize for this error.

